# UAV-Based RGB Imagery for Hokkaido Pumpkin *(Cucurbita max.)* Detection and Yield Estimation

**DOI:** 10.3390/s21010118

**Published:** 2020-12-27

**Authors:** Lucas Wittstruck, Insa Kühling, Dieter Trautz, Maik Kohlbrecher, Thomas Jarmer

**Affiliations:** 1 Remote Sensing Group, Institute of Computer Science, Osnabrück University, 49074 Osnabrück, Germany; thomas.jarmer@uos.de; 2 Agronomy and Crop Science, Kiel University, 24118 Kiel, Germany; kuehling@pflanzenbau.uni-kiel.de; 3 Faculty of Agricultural Sciences and Landscape Architecture, Osnabrück University of Applied Sciences, 49076 Osnabrück, Germany; d.trautz@hs-osnabrueck.de (D.T.); m.kohlbrecher@hs-osnabrueck.de (M.K.)

**Keywords:** remote sensing, drones, random forest, low-cost sensor, winter squash, vegetables, fruit size, fruit weight, Europe

## Abstract

Pumpkins are economically and nutritionally valuable vegetables with increasing popularity and acreage across Europe. Successful commercialization, however, require detailed pre-harvest information about number and weight of the fruits. To get a non-destructive and cost-effective yield estimation, we developed an image processing methodology for high-resolution RGB data from Unmanned aerial vehicle (UAV) and applied this on a Hokkaido pumpkin farmer’s field in North-western Germany. The methodology was implemented in the programming language Python and comprised several steps, including image pre-processing, pixel-based image classification, classification post-processing for single fruit detection, and fruit size and weight quantification. To derive the weight from two-dimensional imagery, we calculated elliptical spheroids from lengths of diameters and heights. The performance of this processes was evaluated by comparison with manually harvested ground-truth samples and cross-checked for misclassification from randomly selected test objects. Errors in classification and fruit geometry could be successfully reduced based on the described processing steps. Additionally, different lighting conditions, as well as shadows, in the image data could be compensated by the proposed methodology. The results revealed a satisfactory detection of 95% (error rate of 5%) from the field sample, as well as a reliable volume and weight estimation with Pearson’s correlation coefficients of 0.83 and 0.84, respectively, from the described ellipsoid approach. The yield was estimated with 1.51 kg m^−2^ corresponding to an average individual fruit weight of 1100 g and an average number of 1.37 pumpkins per m^2^. Moreover, spatial distribution of aggregated fruit densities and weights were calculated to assess in-field optimization potential for agronomic management as demonstrated between a shaded edge compared to the rest of the field. The proposed approach provides the Hokkaido producer useful information for more targeted pre-harvest marketing strategies, since most food retailers request homogeneous lots within prescribed size or weight classes.

## 1. Introduction

**Figure 1:**
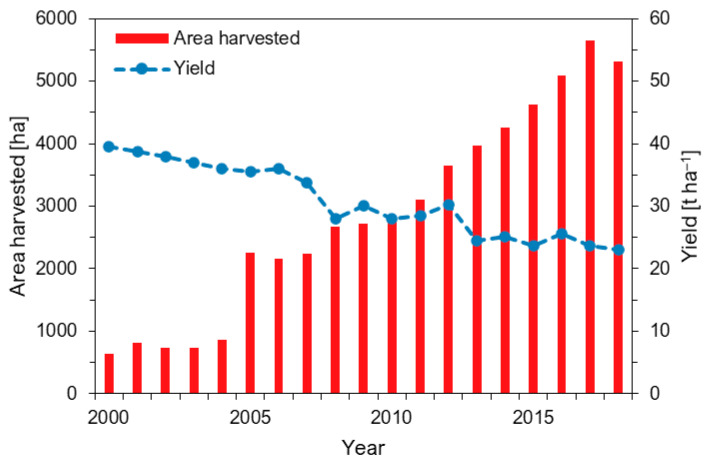
Development of cultivation area and yield in Germany (aggregated data on pumpkin, squash and gourds). Data source: Reference [1].

Pumpkins (*Cucurbita* spp.) are produced on 2 million ha of land worldwide and becoming increasingly popular across Europe [1]. In Germany, the harvested area steadily increased over the last two decades and reached more than 5000 ha in 2018 (Figure 1). Whereas the global yield was on a constant level of about 13.3 t ha^−1^, in Germany, yields are on a slight decrease was observed [1]. This could be due to a shift towards more consumer-oriented production of smaller fruits (<1.5 kg).

The origin of pumpkin domestication was in the Americas, where several *Cucurbita* species were cultivated in the pre-Columbian era; together with maize, they were later brought to Europe [2]. Breeding progress for earliness and adaptation to temperate climate helped to spread the cultivation across higher latitudes. *Curcubita* has manifold forms and properties, and the very popular “Hokkaido” pumpkins belong to the *Curcubita maxima* species within the “Hubbard” varietal group [2]. Whereas, in Europe, only a few species are produced for professional food retailing, most of the manifold pumpkins (or winter squash) are cultivated by private gardeners and in developing countries by local farmers from landraces and saved seeds.

There are two main directions of cultivation: for human consumption of the whole plant or for high-quality oil production (from the seeds), as well as a smaller share as medical plants due to their secondary ingredients (e.g., high amount of carotenoids) [2,3]. The seeds are a high-energy source and known as snack in some regions of the world [4]. The production for European food retail is focusing on small individual fruits since about 0.5 to 1.5 kg. Large pumpkins (>2.2 kg) can successfully be commercialized around Halloween for carving.

Due to a more diverse fruit development in pumpkin fields compared to crops planted in higher stand densities, precise yield predictions are difficult but of great interest for logistic planning and marketing activities. Non-destructive pre-harvest yield estimations with detailed information about size, weight and number of fruits can help farmers to improve their commercialization strategies. Unmanned aerial vehicle (UAV)-based remote sensing approaches are a cost-effective way to gain such information.

In recent years, high-resolution UAV data have become an import source for many agricultural applications, such as plant detection [5,6], plant monitoring [7,8], or yield prediction [9,10]. Especially, as drones and basic RGB camera systems have been getting more affordable, the use of drone systems has increased in the agricultural sector.

Current studies have demonstrated the suitability of UAV data to detect and count fruits for yield estimation, including strawberries [11], apples and oranges [12], citrus fruits [13], or melons [14]. However, from our state of knowledge, almost no studies are available on the detection of pumpkin fruits for yield estimations from UAV data. The main challenges in the above-mentioned papers were related to the detection of single fruits which overlap [11] or were partially hidden under leaves [13]. To overcome those restrictions, the studies used techniques from the currently very successful field of deep learning, allowing the shape of a fruit to be learned using a neural network. However, these models usually require a large amount of training data to outperform classical methods [15] and are very computationally expensive to train.

The aim of this study was to develop a pipeline to detect and count Hokkaido fruits for yield prediction using high resolution UAV RGB imagery. Therefore, we used classical methods from the field of machine learning and computer vision, which have been successfully applied in object detection tasks [10,16]. In order to address the issue of overlapping and close-by fruits, we implemented a simple conditional and thresholding strategy to split those fruits. For shape reconstruction, we used geometric features. Besides this, the estimated number and yield distribution of the fruits within the study area was investigated and evaluated to derive agronomic implications.

## 2. Materials and Methods

### 2.1. Field Management

**Figure 2:**
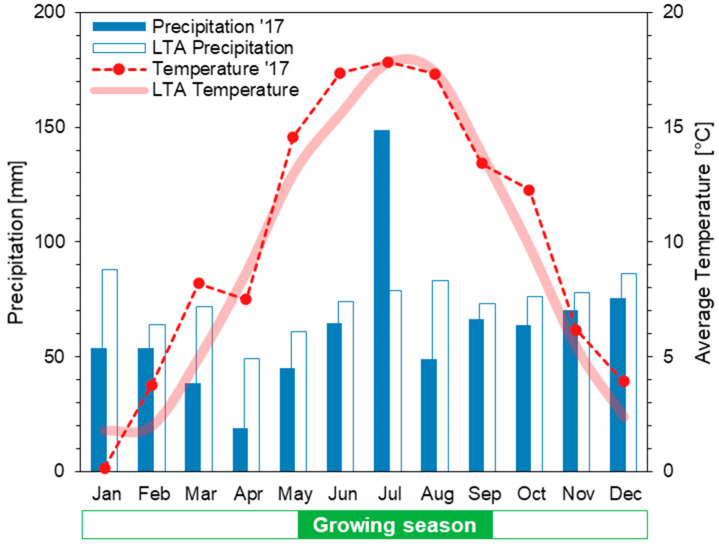
Climate diagram with long-term average values (LTA: 1981–2010) compared to the year 2017 for monthly precipitation and mean air temperatures. Data source: Reference [19].

The experimental site was located on a 0.25 ha field in North-western Germany (52.33° N, 7.97° E WGS84, 65 m asl) close to Osnabrück on a Stagnic Cambisol soil type, following the World Reference Base (WRB) classification [17] with low to medium yield potential. The climate is characterized as temperate oceanic (Cfb according to Köppen-Geiger classification) [18] with a mean annual air temperature of 9.4 °C and 883 mm annual precipitation. The experimental growing season received average precipitation but with a considerably uneven distribution, and temperatures were 1.6 °C above the long-term average (Figure 2).

The previous crop on the field was maize, and all agronomic operations are listed in Table 1. The Hokkaido pumpkins (*Cucurbita max.* Duchesne cv. Uchiki Kuri) were planted as seedlings in growth stage 12 according to BBCH [20] with a semi-automated planting machine in mid-May. Harvest took place at maturity after 130 days.

### 2.2. UAV Image Data

High-resolution UAV image data were acquired on 23 June 2017 under cloudless weather conditions to ensure similar illumination conditions. For the aerial survey, a DJI Phantom 4 was used, which features an on-board RGB camera with a sensor resolution of 12 megapixels and a focal length of 5.74 mm. A flight altitude of 46 m above ground level was chosen. This allowed a spatial resolution of 1 cm and an overall flight time of about 7 min to cover the study area.

For georeferencing, ten field targets were placed evenly in the study area, which were used as ground control points (GCP) in the post-processing step. The target centers were located using a differential GPS (bi-frequency GNSS receiver) based on the German SAPOS correction service for precise positioning in Real-Time Kinematic (RTK) mode.

The flight was conducted at noon so that shadowing was reduced to a minimum. During the flight, we used GPS and GLONASS positioning (p-mode) for platform stability. The camera was oriented vertically using the integrated three-axis gimbal. A total of 106 images were acquired with a side and front overlap of 75%, respectively.

### 2.3. Field Sampling

After the UAV flight, a total of 100 randomly selected Hokkaido pumpkins were measured for diameter and height, and the exact position of each pumpkin fruit was located by differential GPS. The diameter refers to the largest horizontal extension of the pumpkin, whereas the height is determined by the distance between the stem and the blossom end. During harvest, which was carried out a few days after the flight, another 40 pumpkins were measured following the same collection procedure. For yield estimation, we also determined the weights of these pumpkin fruits.

### 2.4. Methodology for Fruit Identification

**Figure 3:**
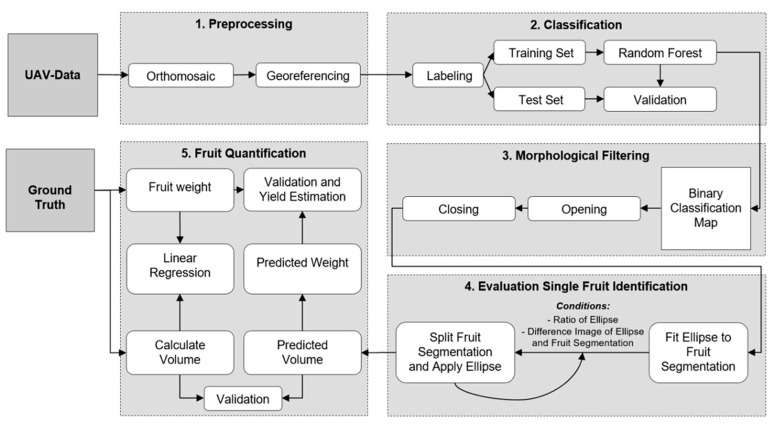
Workflow of the proposed methodology.

The methodology of this work consisted of numerous steps, including image pre-processing (1), pixel-based image classification (2), classification post-processing for single fruit detection (3–4), and fruit quantification (5). All steps of the methodology were implemented in the programming language Python (version 3.7). An overview of the proposed pipeline is illustrated in Figure 3.

In the first step, we created an orthophoto mosaic from the collected UAV images based on photogrammetric processing using the Agisoft Metashape software (Version 1.5.3). In addition, the image was georeferenced using the located in-field targets. Then, we processed the collected UAV images with the Agisoft Metashape software (Version 1.5.3), including image stitching and georeferentiation using in-field targets. For fruit detection and counting, a classification approach was initially used. Therefore, representative pixels of the classes *fruit* and *non-fruit* were manually digitized from the generated orthomosaic. The samples were then divided equally into a training and test set. For each class, 1000 pixels were randomly selected from the test data to evaluate the classification algorithm.

The pixel-based classification of the Hokkaido fruits was based on a binary random forest (RF) algorithm, which consists of large number of decision trees, whose individual results are combined into a single classification result. Since multiple decision trees are used, this approach is considered to be robust against overfitting, as well as unbalanced class sizes [21]. The RF enhances common decision trees using the methods of bagging and random feature selection. The first implies that each decision tree is assigned only to a random selection of training data, whereas the random feature selection reduces the number of input variables randomly [22]. The classification relies on the assignment probabilities of all trees, which are determined by a majority vote. This means that a certain input is assigned to that class, which is predicted by most of the trees.

In this study, the RF classifier was instantiated with a total of 500 trees, since studies have shown that the error term remains nearly constant with this number of trees [23]. To separate the classes within a decision tree, we used the Gini Index, which measures the average gain of class purity by splits of a given variable [24]. The accuracy assessment of the classification model was based on the independent test data. The classification quality was determined using a confusion matrix, including overall accuracy (OA), producer’s accuracy (PA), and user’s accuracy (UA).

In order to overcome misclassifications in the binary classification map, we applied a sequence of morphological filters. The idea behind this approach was to remove isolated pixel with high uncertainty, as well as to increase the homogeneity of the segmented fruit objects in shape and size. Morphological filters are Boolean filters, which are very effective for smoothing binary images [25]. In this context, two basic morphological operators are often used: *erosion* and *dilation*. The *dilation* filter expands the size of an object by adding pixels at the object boundary, which also allows gaps within objects to be closed. In comparison, the *erosion* filter reduces the size of the foreground. Therefore, objects of small size are removed, and object boundaries are sharpened [26]. Both operations produce a homogeneous appearance of objects, depending on the shape of the object and the given filter size. However, the application of this operators also leads to a change in object size. To keep the approximated object sizes, the operators o*pening* and c*losing* are often applied, which uses the operators e*rosion* and *dilation* in sequence. *Opening* is defined as the application of *erosion*, followed by a *dilation*. *Closing* is determined as *dilation*, followed by *erosion*. *Opening* and *closing* have the same smoothing properties as *erosion* and *dilation,* respectively [25]. In this work, we initially applied the *opening* operation to eliminate classification noise, followed by *closing* for smoothing the remaining objects. For the morphological filtering, a kernel size of 3 × 3 pixel was specified.

Even with the application of morphological filtering, the derivation of the correct shape and size of the segmented Hokkaido fruits can be limited if they are partially covered by leaves or stems. To reconstruct the actual shape of the segmented fruits, the geometric form of an ellipse was applied to every segmented object.

The ellipse was chosen because the Hokkaido fruit is normally round-shaped, and its height is usually smaller compared to its diameter. This results in an oval shape, which can be approximated by an ellipse. To apply the ellipses to the segmented objects, every object had to be identified in the image first. This was accomplished by using the method *findContours* by Suzuki et al. [27] of the open-source computer vision library (OpenCV). In the next step, for every fruit object, an ellipse was approximated using the *fitEllipse* algorithm. This function uses the principle of least squares to fit an ellipse to a selection of points on a given two-dimensional plane [28].

**Figure 4:**
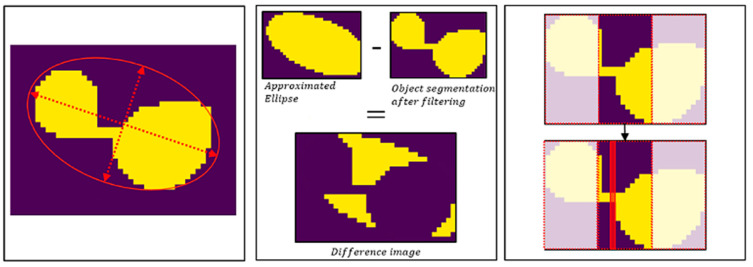
Illustration of the tested conditions for object splitting (left and middle), as well as the implementation for multi-fruit object separation.

Based on the segmentation steps presented, nearby fruits could be classified as joint objects. For the quantification of the Hokkaido fruits, it was essential to split an object accordingly to the number of fruits it represents. In order to select those objects, two conditions were tested: Firstly, the aspect ratios of the applied ellipses were calculated. It was assumed that several fruits within an ellipse result in a stretched geometry, leading to an increased aspect ratio, as illustrated in Figure 4 (left). Secondly, the difference between the fruit ellipses and their corresponding object segmentation after the morphological filtering was calculated. Here, the approximation errors of the ellipses compared to the more precise object segmentations should be highlighted to reveal spaces between the nearby fruits, as shown in Figure 4 (middle). For both conditions, threshold values were applied to identify objects with multiple fruits.

To separate the identified objects, a bounding box was placed around these segmented objects before the ellipse has been applied (Figure 4, right). Then, the object was divided into three equally sized areas, which were always oriented parallel to the shorter length of the bounding box. Within the central area, a separation of the considered object was performed where the object had the lowest extension. After splitting, for every new generated object, an ellipse was approximated, as presented before. For separation of objects that represented more than two fruits, the above steps were repeated iteratively as long as the tested conditions matched a considered object. After this step, the total number of pumpkins in the field could be counted.

**Figure 5:**
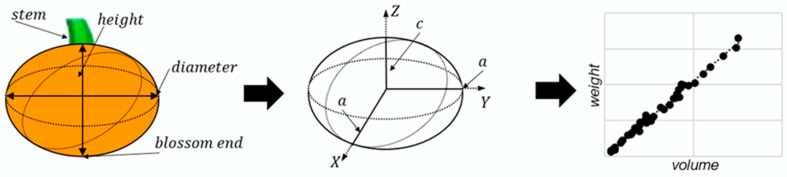
Two-dimensional representation of the pumpkin fruits and its extension in height and diameter (left), volume calculation using shape of spheroid (middle), and empirical linear regression for weight estimation of detected fruits from volume (right).

Besides the counting of fruits, this study aimed to derive the weights of Hokkaido fruits for yield prediction. However, the fruit weights could not be determined directly from the UAV data, due to their two-dimensional representation in the orthomosaic. Nevertheless, we assumed that the fruit weight is strongly related to the fruit volume, which can be approximated by a 3D oblate spheroid shape. An oblate spheroid is defined by a minor axis (c) and two major axes (a), resulting in symmetrical form with flattened height. This form is very similar to the shape of a Hokkaido, which is mostly round-shaped and squashed from above (Figure 5). 

For volume approximation, the diameters and heights were determined of each fitted ellipse in the image and correlated with the major and minor axes of the spheroid using the semi-height and semi-diameter (Equation (1)).

(1) $$V_{spheroid}=\frac{4\pi }{3}\;*a^{2}*c\;\;V_{pumpkin}=\frac{4\pi }{3}\;*{\left(\frac{diameter}{2}\right)}^{2}\;*\frac{height}{2}\;.$$

For this, we defined the longer axis of the ellipse to be the diameter of a fruit, whereas the shorter axis was set as height. As the Hokkaido pumpkins predominantly lie sideways, we assumed that the height and the diameter could be plausibly derived from the image data. For the weight estimation, we calculated an empirical linear regression using the weights of the 40 harvested pumpkins and used the calculated regression equation to estimate the weight from the calculated volume of each generated ellipse. The performance of the regression model was validated using the coefficient of determination (r^2^).

The evaluation of the algorithm for fruit detection, as well as the estimation of fruit volumes and weights from UAV imagery, was performed through the comparison between the measured pumpkin fruits in the field and the corresponding fruits found in the image. For volume and weight estimation, the performance was investigated using the Pearson correlation coefficient. In order to evaluate incorrectly identified fruits in the UAV image, we used a random number generator (RNG) to select 200 objects from the orthoimage, which were then manually checked by human for accuracy.

## 3. Results

### 3.1. Descriptive Statistics of Field Data

Heights of the pumpkin fruits were measured in the range 6.5–19.0 cm (mean: 12.4 cm). Measurements of the pumpkin fruits diameters provided very similar values with slightly higher maximum (21.0 cm) and a mean of 13.8 cm, but pumpkin fruits with very low diameter of 6.0–7.0 cm were slightly overrepresented in the collected data set. The difference in mean of height and diameter is typical due to the slightly flattened shape of Hokkaido pumpkins. Weight varied over a wide range (142–3011 g; mean: 1.187 g), which was indicated by a standard deviation (SD) of 776.2 g. In case of pumpkin fruits weight, we sampled a high number of very light fruits with eight out of 40 pumpkins weighted less than 420 g. Detailed descriptive statistics of the investigated fruits are summarized in Table 2.

### 3.2. Accuracy Assessment of Random Forest Classification

With an overall accuracy of 94.7% most of the pixel were correctly identified. However, differences in accuracy were found between the two classes tested. With a PA of 97.3%, *non-fruit* pixels could be classified particularly successfully. Higher estimation errors occurred for the *fruit* class, where an PA of 92.1% was achieved. For the user’s accuracy, the results of the considered classes were reverse. In this way, a UA of 92.5% for the *non-fruit* class could be achieved, whereas the UA for the pumpkin class reached 97.3%.

### 3.3. Mapping of the Estimated Hookaido Pumpkins

**Figure 6:**
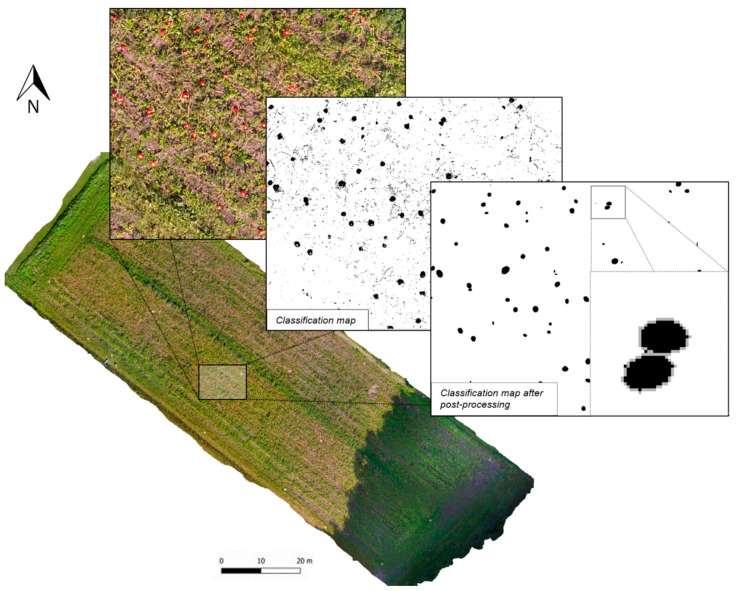
Orthophoto with selected classification steps during fruit detection procedure.

Figure 6 shows the generated orthophoto, as well as a subset, of the classification map before and after the post-processing steps. In the UAV imagery, it can be seen that the investigated field was divided into an illuminated and a shaded region. Because of this, the fruit colors appeared very different in the orthophoto. In the illuminated part of the image, the fruits showed a very light reddish coloring. Due to the direct sunlight, the fruits were partially overexposed in many cases, resulting in a bright spot. Additionally, shadows appeared more frequently in this area of the image. In the shaded area, the fruits were evenly colored in an intense red, so that they could be clearly differentiated from the soil and the plants. In many cases, the fruits were only partially visible, as they were covered by leaves or stems.

Despite different representations of the Hokkaido fruits in the image, the majority of the fruits was correctly identified based on the initial RF classification. Especially, in the shaded area of the image, fruits were classified very precisely in size and shape. Fruits in the illuminated region were recognized with a similar accuracy, although some fruits, which were partially overexposed, revealed gaps. Besides this, bright plant stems were often incorrectly recognized as Hokkaido fruits. In comparison, these classification errors did not appear in the shaded area. Frequently, nearby pumpkins were merged into single segmented objects.

The classification map after the post-processing steps provided a significant improvement in the classification performance. Therefore, misclassifications could be removed, and gaps within the pumpkin objects, as well as errors of the pumpkin shape, have been decreased substantially. Furthermore, joined objects, which represent multiple pumpkin fruits, could be separated successfully in most of the cases. In only a very few examples, single segmented fruits were separated.

### 3.4. Quantification of Hokkaido Fruits

The presented mapping result revealed a high detection accuracy, which can be statistically confirmed, as the algorithm was able to detect 95% of the located and measured fruits from our field sampling. Additionally, it was shown that, from a sample of 200 detected pumpkins, all of them actually corresponded to real pumpkin fruits. 

The regression model to predict the fruits weights from volumes indicated the strong relationship between calculated volume (from Equation (1)) and observed weight. The variance of the measured weights could be explained by more than 99% by the calculated pumpkin fruit volume. Consequently, the pumpkin fruit volume can be assumed as a very good proxy for the fruit weight. 

**Figure 7:**
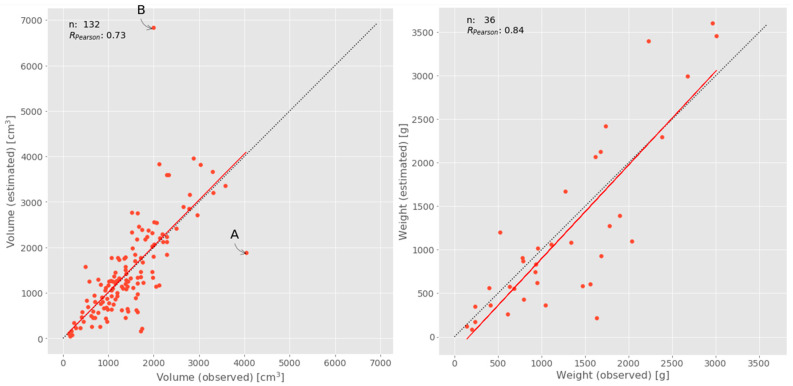
Scatterplot of observed and estimated fruit volume (**left**) and weight (**right**) with 1:1-line (gray) and correlation (red).

**Figure 8:**
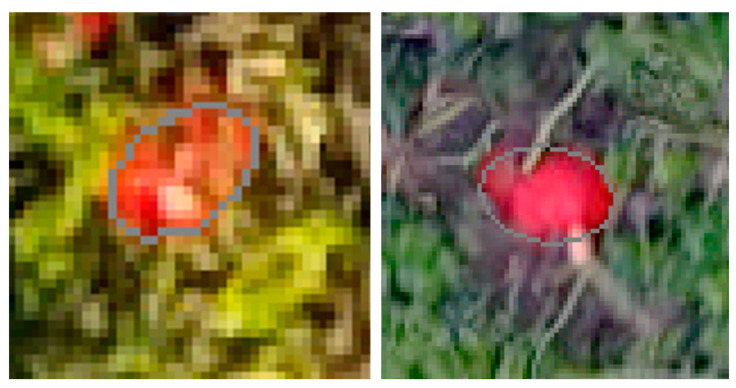
Prediction errors due to overlap by leaves (**left**) and insufficient image mosaicking (**right**).

Figure 7 (left) indicates a reliable relationship between the fruit volume observed and estimated from classified fruits in the image data with a correlation coefficient of 0.736 (significant at 99% level). If looking at the scatterplot in details, obviously, two samples do not follow the general trend. Fruit “A” was observed with a volume of 4040 cm³ but estimated with the much lower volume of 1885 cm^3^. The reason for this mismatch was that this fruit was partly covered by leaves (Figure 8, left). The other conspicuous case was fruit “B” with an observed volume of 1995 cm^3^ but estimated with a volume more than three times of the observed one (6839 cm^2^). In this case, insufficient image mosaicking was responsible for this “huge” fruit explaining the extremely high volume (Figure 8, right). If these two obviously erroneous cases were excluded from analysis, the correlation increases substantially to 0.826 (significant at 99% level).

Correlation between observed and estimated weight of fruits was found to be highly significant at 99% level with an r of 0.844. Although a certain scattering of data was observed, no noticeable outlier was identified (Figure 7, right).

The predicted fruit weights in the image were divided in weight classes, according to Reference [29]. Table 3 summarizes the total number of saleable (72.5%) and the share of non-marketable fruits from the mapping and classification procedures. Small pumpkins below 500 g can be utilized as animal feed.

**Figure 9:**
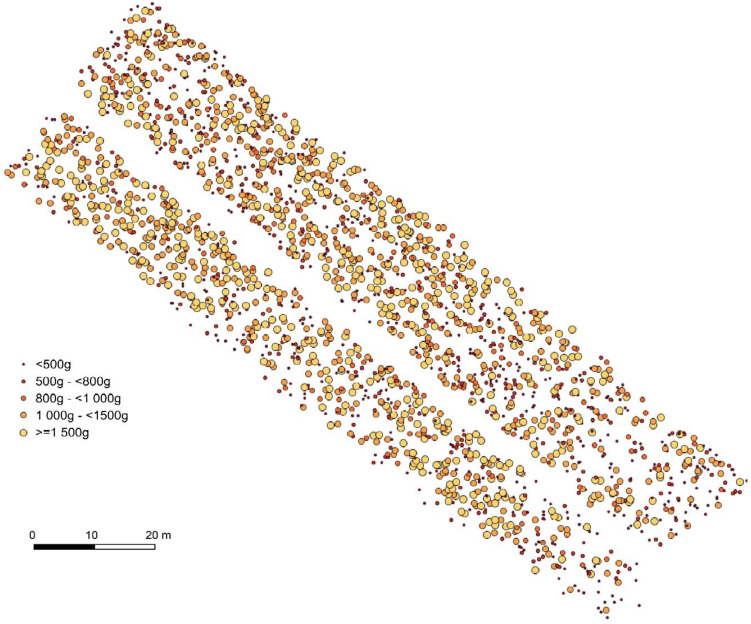
Field distribution of pumpkin fruits by weight classes.

Figure 9 shows the spatial distribution of pumpkins by weight classes according to the market practices with a gradient from more and heavier fruits to less and lighter pumpkins towards the south-eastern part of the field. 

**Figure 10:**
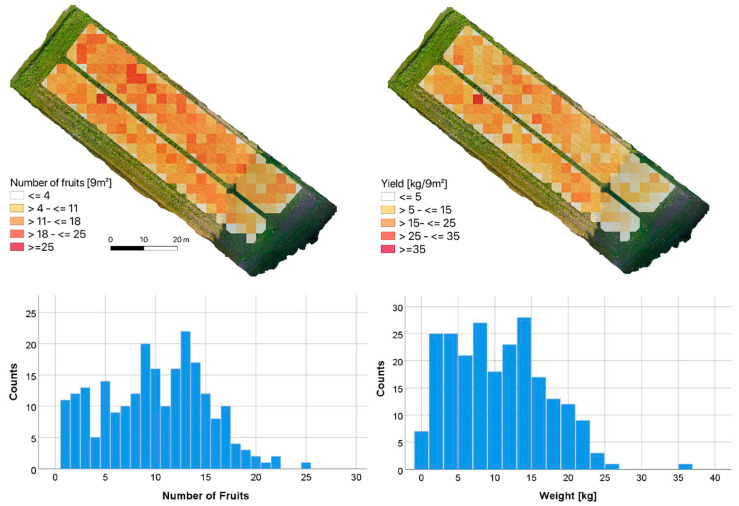
Spatial pattern of 9 × 9 m grid cells and frequency distributions for number of fruits (left) and weights (right).

In Figure 10, an aggregated in-field distribution of number and weight per 3 × 3 m is presented. The number of fruits in the cells varied between one fruit and 25 fruits (Figure 10 left). The mean number of fruits for the entire field was determined with 1.37 fruits (SD: 1.19) per m^2^. A similar pattern of weight classes was derived from Figure 10 (right) with pumpkin weights ranging from less than one kg to more than 35 kg in the maximum. However, only one cell provided a weight higher than 26 kg. In relation to the field size, a mean weight of 1.51 kg m^−2^ (SD: 1.74) was estimated. Going into detail, one cell provided both the highest number of pumpkin fruits (25 per 9 m^2^), as well as highest yield (35.7 kg per 9 m^2^).

Looking at the south-eastern part of the field exhibits a different pattern compared to the rest of the field. In this area, the number of fruits, as well as the yield, were substantially below the rest of the field. This disparity shows the impact of the shading at the south-eastern edge with lower area-scaled average values. Besides this, no spatial pattern for the investigated field could be observed, neither for the number of fruits nor the yield.

Linking the number of fruits per cell to the predicted weight provided a substantial relationship with an r of 0.882 (significant at 99% level). Obviously, the number of fruits within a 3 × 3 m cell is highly positively correlated with the yield per cell.

## 4. Discussion

Many investigations described approaches for yield estimation from UAV-based images for crops, like cereals or oilseed rape, that form yield from stands with uniform single plants [30,31,32]. Those concepts often use total aboveground biomass and derive grain yield by known or estimated harvest index. In addition, applications for orchards were described for estimating fruit yield from permacultures, like apple or citrus, where the trees remain on the field [12,13]. For vegetables, stand height or biomass-based applications were described for eggplants, tomato, and cabbage under tropical conditions in India [33]. Kalantar [14] presented a successful individual fruit detection for yield estimation in a melon field where leave senescence started before harvest. Here, we presented a novel method for precise yield prediction based on single fruit identification, including size and weight classification, that allows more detailed harvest determination from a vegetable field with Hokkaido pumpkins between living leaves and stems.

### 4.1. Methodologic Performance

The pixel-based classification results using the RF classifier revealed high accuracies for both classes. However, it was found that *fruit* pixels were more often mistakenly classified as *non-fruit* than vice versa. One reason can be the high heterogeneity of this class, which includes different surfaces in various lightning conditions. In comparison, a very high user’s accuracy was achieved for the *fruit* class. This indicates that pixels of this class have a very high probability of belonging to this class [34], which is important for fruit counting as it reduces the number of wrong identified pumpkins as shown.

The mapping of the estimated pumpkin fruits revealed that the detection of single pumpkin fruits, as well as the approximation of their shapes, was achieved with high accuracies. RF classifications are described to achieve good accuracies for fruit detection [35], but the challenge increases in images from natural environments, as in our case. Regarding the RF classification approach, misclassifications in the illuminated field region between fruits with high reflectance (bright spot) and plant stems was limited, due to the similar color representation in the image, whereas other studies aimed on an additional shadow-removal step during the post-processing to reduce classification errors [36]. In our example, in the shaded area, noticeably fewer pixels were incorrectly assigned, which can be explained by the distinct color difference between the reddish fruits and the dark appearance of the soil and plants. The morphological filtering led to improved classification results. So, misclassified pixel of plant stems could be eliminated, and gaps within the segmented objects could be closed. This was possible as wrong classified pixel often occurred isolated or in a very narrow extend, which allowed them to be differentiated from the usually larger fruits.

The separation of neighboring pumpkin fruits was successfully applied in most cases. This issue usually poses a great challenge in single fruit detection [12,37]. A few errors were recognized where blurring was present in the image. Therefore, contours of individual fruits could not be precisely approximated by the algorithm. 

The estimation of the fruit volumes and weights from the UAV image showed reliable correlations to the observed data. This confirms the positive results for single fruit detection and shape estimation based on the implemented algorithm. Uncertainties in prediction could be attributed to errors in image mosaicking and the occlusions from leaves and stems. The second issue has been already discussed in similar studies [12,13]. A further limitation in the prediction is given if pumpkins are growing vertically in the field because then the height of the fruit cannot be estimated from the top view image. However, this could only be observed for a small number of the pumpkins harvested.

### 4.2. Agronomic Consequences

Compared to a field survey from Hokkaido cultivation of the same cultivar in Germany [29], the average yield of 1.51 kg m^−2^ with an average number of 1.37 pumpkins per m^2^ was lower (2 fruits m^−2^, 1.91 kg m^−2^). Even though the plant density of 1 seedling m^−2^ was the same, these differences can be explained by higher fertilization (∆ N 45 kg) and drip irrigation versus rainfed water supply in our experiment. Besides lower yield, we observed a more equal fruit distribution among the weight classes compared to Reinisch and Sauer [29]. The share of marketable fruits from our detection was a little lower than from trials in North-Eastern Germany with the same cultivar, where around 80% of the harvested pumpkins reached a weight >500 g but also with a higher fertilization level (∆ N 65 kg) [38]. From this trial, a slightly lower average fruit weight (950 g compared to 990 g) was reported, which indicated a more homogenous size distribution. The relatively high share of undersize fruits is typical for the observed cultivar Uchiki Kuri and can be seen as a genetic trade-off from the production goal of small to medium pumpkins [29,39] but may be compensated by better N supply [29].

Additionally, the lower weight estimations compared to other studies can be explained through difficulties during the mapping and classification procedure, since some fruits were partially covered by leaves or stems, which led to slight underestimations, specifically for the higher share of smaller pumpkins (Figure 9 and Figure 10).

Interestingly, investigations on optimal stand densities revealed higher yield tendencies towards less plants per m^2^, which was not reflected in our within-field variation of the yield distribution [38]. From Figure 10, no clear pattern showed advantages from more space at the borders of the two patches. The only clear gradient was towards the south-eastern, shaded part of the field (Figure 9 and Figure 10), where less and smaller pumpkins were classified. Since radiation is the main driver for photosynthesis and, consequently, dry matter accumulation in fruits, this explains very plausibly the reduced pumpkin development close to the shading trees at that edge of the field [40]. Other resource use competition, specifically for water, is of minor importance due to lower temperatures, thus resulting less evapotranspiration from the shading trees. Hence, the producer can implement this knowledge for future pumpkin cultivation and increase seedling density in this part of the field in the next rotation.

### 4.3. Future Perspectives

To optimize the proposed methodology, future studies should aim to detect and reconstruct fruit shape, when they are hidden by leaves or stems. The use of multispectral and thermal sensors could be useful for this purpose, as differences in reflectance between plant components can be assumed. In addition, the methodology should be tested under further field and flight conditions (e.g., change of flight altitude, seasonality, slope of field).

Further agronomic applications from such UAV imagery processing might be in-field differentiated sustainability evaluations through deriving, e.g., radiation use efficiency or water use efficiency. In general, the described methodology is transferable to other fruits or vegetables with above ground harvest products to solve similar logistical challenges as described for Hokkaido pumpkins.

## 5. Conclusions

In this paper, we proposed a method to detect Hokkaido fruits and estimate their volumes and weights for yield prediction using high resolution UAV imagery. The results showed that most of the fruits could be satisfactorily identified. Misclassifications and errors in fruit geometry could be successfully reduced based on the processing steps presented. In addition, different lighting conditions, as well as shadows, in the image data could be compensated by the proposed methodology.

The volumes and weights of the pumpkins could be derived from the image data with high precision, allowing more targeted pre-harvest commercialization strategies for farmers. Since most food retailers request homogeneous lots within prescribed size or weight classes, producers can improve their sales negotiations with better knowledge of classified sales volume.

## Figures and Tables

**Table 1:** Agronomic management during the cropping season 2017. DAP: days after planting.

Date	DAP	Management
30 March	−48	Tillage: Primary soil cultivation by moldboard plough (25 cm depth)
15 May	−2	Fertilization: 60 kg N ha^−1^, 60 kg P ha^−1^, 60 kg K ha^−1^
16 May	−1	Tillage: Seedbed preparation by cultivator with crumbler roller
17 May	0	Planting: Cultivar Uchiki Kuri, 10.000 plants ha^−1^, 2 m row width
24 May	7	Weed regulation: once a week, 5 times Between rows by harrow, in row manually
7 June	21	Fertilization: 15 kg N ha^−1^, 15 kg P ha^−1^, 15 kg K ha^−1^
24 September	130	Harvest

**Table 2:** Descriptive statistics of pumpkin fruits (n: sample size).

	n	Minimum	Maximum	Mean	SD
Height (cm)	140	6.5	19.0	12.4	2.6
Diameter (cm)	140	6.0	21.0	13.8	3.0
Weight (g)	40	142.0	3011.0	1187.0	776.2

**Table 3:** Distribution of Hokkaido fruits within the weight classes for food retailing.

Weight Class (kg)	Classification Data	Percentage
<0.5 *	628	27.5
0.5–0.8	351	15.3
0.8–1.0	204	8.9
1.0–1.5	535	23.4
≥1.5	568	24.8
Sum	2286	99.9

* non-marketable → forage.

## Data Availability

Data is contained within the article
